# Feasibility and Initial Outcomes of Telesurgery in Urology: a Systematic Review of the Literature

**DOI:** 10.1590/S1677-5538.IBJU.2024.0494

**Published:** 2025-01-21

**Authors:** Sávio Valadares Ferreira, Murilo Henrique Sugai, Guilherme Corrêa do Nascimento, Antonino Caetano de Souza, Gustavo Colombo Cabrini, Fernando Martins Rodrigues, Cleverson Luiz Rocha D’Avila, Geovanne Furtado Souza, Ricardo Vieira Zerati, Miguel Zerati

**Affiliations:** 1 Instituto de Urologia e Nefrologia São José do Rio Preto SP Brasil Instituto de Urologia e Nefrologia, São José do Rio Preto, SP, Brasil; 2 Hospital Padre Albino Catanduva SP Brasil Hospital Padre Albino, Catanduva, SP, Brasil

**Keywords:** Systematic Review [Publication Type], Robotics, Urology

## Abstract

**Introduction:**

Telesurgery allows the procedures to be carried out over long distances, however due to lack of data, its feasibility has not been consolidated yet. Since it is a promising modality, it is important to illustrate the current scenario on this subject.

**Objective:**

To review the literature aiming at the surgical success rate as a primary objective, and secondly, the most important patient outcomes and the network system.

**Materials and Methods:**

In June 2024, we followed PRISMA guidelines to research trials on urological robotic surgery in humans. We used as exclusion criteria: editorials, specialist's opinions, tele-mentoring, tele-training, small procedures, non-remote surgeries, absence of interest outcomes, telesurgeries in non-humans or in cadaver.

**Results:**

Five hundred and ninety eight studies were identified with peer review and a third reviewer for divergencies, both directed by previously established inclusion and exclusion criteria, selecting 6 studies after the exclusions. We found 54 patients who underwent urological telesurgeries; all of them were accomplished with no complications or need for conversion to open surgery. Almost all the procedures were carried out in China (98.14%) and the most used robotic model was MicroHand S (83.33%). Nephrectomy was the procedure of choice (57%). Mean surgical time was 66.2 (IQR) 56.6 minutes. Intraoperative bleeding time was 68.6 ± 76.7 milliliters. Hospital stay was 5.5 (IQR) 5 days. The distance between main surgeon and the patient was between 2,581.5 (IQR) 2,871 kilometers. 5G network was used the most (98.14%). The total network latency time was 176 (IQR) 10.9 milliseconds.

**Conclusion:**

Despite its limitations, there was evidence demonstrating that robotic surgery in the genitourinary system is safe and feasible, however it is a subject that must be well discussed, and further studies must be carried out.

## INTRODUCTION

Laparoscopic surgery was at first a reason for jokes ("Mickey Mouse surgery" and "small brain-small incision.") and presented great resistance for its acceptance, since, at that time they couldn't see its huge potential. However, as time went by and due to its excellent results, it became very well accepted (1). In times of war, the military personnel tried remote medical care as an alternative to the difficulties they encountered (2). Due to COVID 19 Pandemics there was a need to communicate without close contact, this way, this concept gained momentum and intensified telemedicine in medical practice (3, 4). With the arrival of new robotic platforms and improvement in telecommunications, the association between these events became inevitable, allowing a transatlantic telesurgery to be performed successfully, becoming a landmark at that time and until today it still impacts current discussions (5–7).

In general, the definitions found show that telemedicine may be defined as an interaction among multimedia, telecommunications and robotic technologies to offer clinical or surgical care. In such telesurgery context, when there is a surgeon with active control operating the surgical instruments of a robot, the surgeon and patient don't necessarily have to be in the same place (8–10). The concept of surgical telepresence has changed the paradigms and generated major developments in laparoscopic surgery, allowing the introduction of robotic systems in daily routine. The first surgical prototype approved by the FDA was the Automated Endoscopic System for Optimal Positioning AESOP^®^. Then, other platforms also gained ground and currently the one that is mostly used is a model called "Da Vinci" produced by Intuitive Surgical (2, 6).

Among surgical specialties, urology stood out, and we could follow its technological evolution closely. Despite the advances in telesurgery, access to this resource is still restricted, although promising, it may generate several benefits to the world's population, especially in remote areas where specialized medical care services are not available (10, 11). In such context, it is worth reviewing the literature to illustrate the current scenario on the subject.

## MATERIALS AND METHODS

### Protocol and Registration

**Figure 1:**
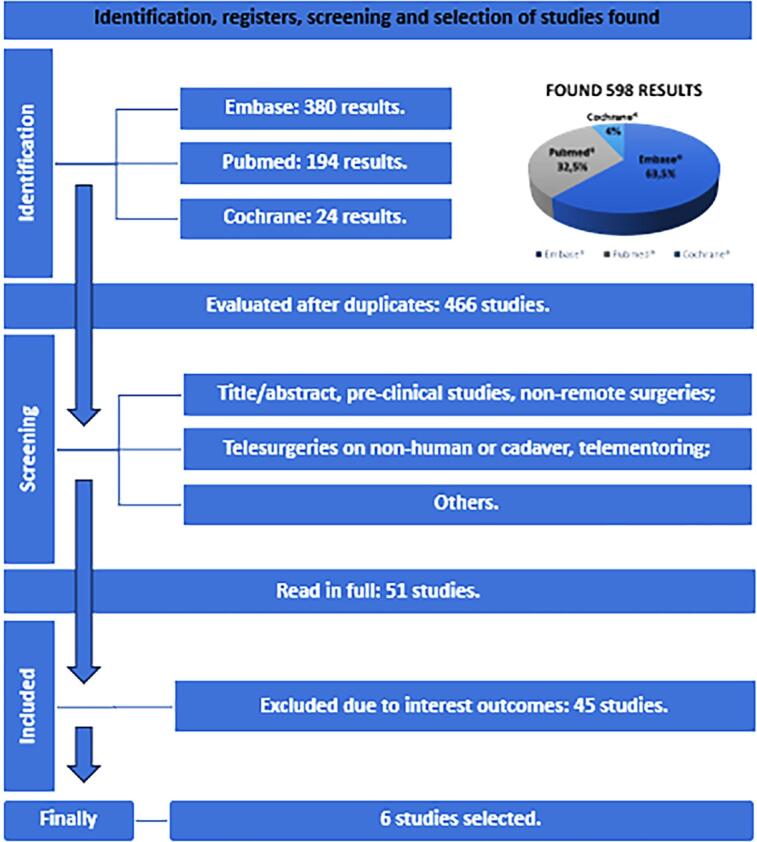
PRISMA diagram of study screening and selection

The guidelines called "Preferred Reporting Items for Systematic Reviews and Meta-Analysis" (PRISMA) were followed to carry out research in June 2024 combining terms on the subject with Boolean operators (Figure-1) in PubMed, Embase and Cochrane platforms to identify trials registered up to that period. We structured the study question based on PICO strategy (P: Patients who underwent urologic robotic telesurgery; I: Remotely performed surgery; C: With no comparisons with other methods; O: Primary: Surgical success rate. Secondary: Main patient clinical outcomes and the network system), registering a protocol at International Prospective Register of Systematic Reviews (PROSPERO; ID CRD42024557337) released for online access. Search strategies, as well as figures and tables will be made available.

### Eligibility criteria

Inclusion criteria were randomized or non - randomized, with patients who underwent urinary tract robotic telesurgery and who reported any of the outcomes of interest. Editorials, expert opinion, tele-mentoring, tele-training, minor procedures, non-remote surgeries, absence of outcomes of interest, telesurgeries on non- humans or performed on cadavers were excluded.

### Trials Selection

The trials found were distributed in the Zotero^®^ 6.0.36 program to help with duplicates and initial selection, the latter being carried out by peers (SVF and MHS), in an independent fashion and the divergencies were clarified by another researcher (MZF), both directed by previously established inclusion and exclusion criteria. References from the included trials, previous systematic reviews and meta-analysis were also manually searched for additional trials.

### Data collecting process and risk of bias

After final selection of the trials, data were manually collected and registered in an Excel Table – Microsoft Office Professional Plus^®^ (2019) to organize the results, conversion and basic statistics such as frequencies and proportions, mean ± standard deviation, median with interquartile range (IQR: subtracting the third from the first interquartile interval) according to the need of how the data were reported. The trials were varied and heterogeneous as to the measurements and effect estimates (12) for samples of continuous and or categoric outcomes were acquired with the help of a calculator, available online (https://www.math.hkbu.edu.hk/~tongt/papers/median2mean.html) to detect asymmetries. Besides that, we used JBI tool as critical assessments of the selected studies (13, 14).

### Measures of association and subgroup analysis

The encountered outcomes of interest were worked with the help of an Excel program– Microsoft Office Professional Plus^®^ (2019) for conversion into frequencies and proportions, mean ± standard deviation, median with calculated interquartile range, according to how they were available in selected trials. A subgroup analysis was restricted to the most used surgical robotic surgical model and the type of procedure most frequently performed in the sample.

## RESULTS

### Selection of trials

Five hundred and ninety-eight records of results up to June 2024 were found with 132 duplicates excluded. After the initial analysis was reviewed by peers based on pre-established criteria, 415 studies were excluded with the help of a third investigator, both independently. The remaining 51 studies underwent a detailed analysis and 6 of them were selected (15–20) for final data collection, identifying a total sample of 54 patients who underwent telesurgery of the genitourinary system (Figure-1).

### Trials characteristics

As urologic robotic telesurgery is an innovative modality, we didn't find randomized, multicentric trials, instead, a heterogeneous and small sample of patients who underwent the procedure in highly specialized centers, both represented in Table-1.

**Table 1:** Characteristics and results of the studies.

Author	Study	Sample	Robotic	Surgeon	Patient	Surgical time (min)	Blood (mL)	Hospitalization (days)	Distance (km)	Network	Total Latency (ms)
**Frimberger, et al. 2022 (** **15** **)**	case reports	Woman 46 years old, with cystic renal mass.	AESOP / RCM + PAKY	Baltimore	Munich	120	50	2	8000	ISDN	
**Li, et al. 2023 (** **16** **)**	case series	Total of 15 patients, 8 men, age 58 (IQR) 7.9, both with adrenal tumor.	MicroHand S	Qingdao	Zhucheng, Zibo, Pingyi.	45 (IQR) 22	25.69 ± 17.34		199 (IQR) 167,5	5G	31.5 (IQR) 2.32
**Li, et al. 2023 (** **17** **)**	case series	Total of 29 patients, 15 men, age 63 (IQR) 18, both with kidney tumor	MicroHand S	Qingdao	Shandong, Gansu.	67 (IQR) 21		8 (IQR) 2	187 (IQR) 57	5G	176 (IQR) 5
**Wang, et al. 2024 (** **18** **)**	case series	Total of 6 patients, all men, age 51 (IQR) 38, who presented one of the following pathologies: retrocaval ureter, adrenal tumor, kidney tumor, prostate tumor.	Edge - MP1000	Beijing, Sanya	Sanya, Beijing	65.5 (IQR) 51.5	62.5 ± 76.53	5.5 (IQR) 3	3.000	5G	171.04 (IQR) 4.23
**Yang, et al. 2022 (** **19** **)**	case reports	Man, 71 years old with bladder tumor	MicroHand S	Qingdao	Anshun	300	200	18	2.163	5G	254 (IQR) 12
**Zhou, et al. 2022 (** **20** **)**	case reports	2 men with varicocele.	Tumai	Nanjing	Xinjiang Kezhou	42.5 (IQR) 5	5	3	3.800	5G	130

### Sample results

We found a total of 54 patients that underwent urologic robotic telesurgery, both completely finished without conversion to open procedures or important intraoperative complications. Surgical time was 66.2 (IQR) 56.6 minutes, with intraoperative bleeding of 68.6 ± 76.7 milliliters, being described that, in one of the trials (17), 1 patient presented intraoperative blood transfusion as prophylaxis justified by preoperative laboratory tests results. The period of hospital stay was 5.5 (IQR) 5 days and almost all the procedures were carried out in China (98.14%). The distance between the main surgeon and patient was 2,581.5 (IQR) 2,871.7 kilometers and the most used internet network was 5G (98.14%), with total latency time of 176 (IQR) 10.6 milliseconds (Table-1).

### Subgroup analysis and risk of bias

The subgroups were analyzed according to the initial guidance of PICO question. In this part of the sample, we identified that the most used robotic platform was model MicroHand S. It was used in 83% of the procedures (45 surgeries), mean age of the population was 63 (IQR) 7 years, of which 53.33% were men. Surgical time was 64 (IQR) 127.5 minutes, carried out from a distance of 199 (IQR) 988 kilometers and total latency of 215 (IQR) 39 milliseconds. Then, we realized that nephrectomy was performed in 57% of the samples (31 patients), with mean age of 57.25 (IQR) 5.75 years. 5G connection was used in all patients with total latency of 173.24 (IQR) 2.75 milliseconds, surgical time of 57.7 (IQR) 9.25 minutes and hospital stay of 6 (IQR) 2 days (Table-1).

After data collecting and analysis of estimates of sample mean values, significant asymmetries were not identified in the results. Besides, the results from the performed critical assessment (SVF and MHS) are also attached (Tables 2A-F).

**Table 2A:** Critical appraisal checklist for case reports. (Frimberger, et al. 2022 (15))

JBI checklist questions	Yes	No	Unclear	Not applicable
Were patient's demographic characteristics clearly described?			✓	
Was the patient's history clearly described and presented as a timeline?		✓		
Was the current clinical condition of the patient on presentation clearly described?			✓	
Were diagnostic tests or methods and the results clearly described?	✓			
Was the intervention(s) or treatment procedure(s) clearly described?	✓			
Was the post-intervention clinical condition clearly described?		✓		
Were adverse events (harms) or unanticipated events identified and described?		✓		
Does the case report provide takeaway lessons?	✓			

**Table 2B:** Critical appraisal checklist for case series. (Li, et al. 2023 (16))

JBI checklist questions	Yes	No	Unclear	Not applicable
Were there clear criteria for inclusion in the case series?	✓			
Was the condition measured in a standard, reliable way for all participants included in the case series?	✓			
Were valid methods used for identification of the condition for all participants included in the case series?	✓			
Did the case series have consecutive inclusion of participants?			✓	
Did the case series have complete inclusion of participants?			✓	
Was there clear reporting of the demographics of the participants in the study?	✓			
Was there clear reporting of clinical information of the participants?	✓			
Were the outcomes or follow up results of cases clearly reported?		✓		
Was there clear reporting of the presenting site(s)/clinic(s) demographic information?	✓			
Was statistical analysis appropriate?	✓			

**Table 2C:** Critical appraisal checklist for case series. (Li, et al. 2023 (17))

JBI checklist questions	Yes	No	Unclear	Not applicable
Were there clear criteria for inclusion in the case series?	✓			
Was the condition measured in a standard, reliable way for all participants included in the case series?	✓			
Were valid methods used for identification of the condition for all participants included inthe case series?	✓			
Did the case series have consecutive inclusion of participants?	✓			
Did the case series have complete inclusion of participants?	✓			
Was there clear reporting of the demographics of the participants in the study?	✓			
Was there clear reporting of clinical information of the participants?	✓			
Were the outcomes or follow up results of cases clearly reported?		✓		
Was there clear reporting of the presenting site(s)/clinic(s) demographic information?	✓			
Was statistical analysis appropriate?	✓			

**Table 2D:** Critical appraisal checklist for case series. (Whang, et al. 2024 (18))

JBI checklist questions	Yes	No	Unclear	Not applicable
Were there clear criteria for inclusion in the case series?			✓	
Was the condition measured in a standard, reliable way for all participants included in the case series?	✓			
Were valid methods used for identification of the condition for all participants included inthe case series?	✓			
Did the case series have consecutive inclusion of participants?			✓	
Did the case series have complete inclusion of participants?			✓	
Was there clear reporting of the demographics of the participants in the study?	✓			
Was there clear reporting of clinical information of the participants?	✓			
Were the outcomes or follow up results of cases clearly reported?	✓			
Was there clear reporting of the presenting site(s)/clinic(s) demographic information?	✓			
Was statistical analysis appropriate?	✓			

**Table 2E:** Critical appraisal checklist for case reports. (Yang, et al. 2022 (19))

JBI checklist questions	Yes	No	Unclear	Not applicable
Were patient's demographic characteristics clearly described?	✓			
Was the patient's history clearly described and presented as a timeline?	✓			
Was the current clinical condition of the patient on presentation clearly described?	✓			
Were diagnostic tests or methods and the results clearly described?	✓			
Was the intervention(s) or treatment procedure(s) clearly described?	✓			
Was the post-intervention clinical condition clearly described?			✓	
Were adverse events (harms) or unanticipated events identified and described?			✓	
Does the case report provide takeaway lessons?	✓			

**Table 2F:** Critical appraisal checklist for case reports. (Zhou, et al. 2022 (20))

JBI checklist questions	Yes	No	Unclear	Not applicable
Were patient's demographic characteristics clearly described?			✓	
Was the patient's history clearly described and presented as a timeline?			✓	
Was the current clinical condition of the patient on presentation clearly described?			✓	
Were diagnostic tests or methods and the results clearly described?			✓	
Was the intervention(s) or treatment procedure(s) clearly described?	✓			
Was the post-intervention clinical condition clearly described?	✓			
Were adverse events (harms) or unanticipated events identified and described?			✓	
Does the case report provide takeaway lessons?	✓			

**Search strategy:** (robotic AND nephrectomy OR pyeloplasty OR nephroureterectomy OR cystectomy OR prostatectomy OR lymphadenectomy OR raveil OR ‘ra veil’ OR ‘da vinci’ OR urology) AND (‘tele surgical’ OR telesurgical OR telesurgery OR transcontinental OR ‘telepresence surgery’ OR toumai OR kangduo OR raven OR microport)

## DISCUSSION

Despite the limitations, we found evidence that it is possible to perform urological telesurgery with safety. The available data demonstrate that all remote procedures were concluded with safety without significant complications (Table-1). Infrastructures disparities in health and internet network associated to limitations to new technologies may be a challenge, however, a notable fact is that most of the procedures relied on a type of network that is well available worldwide, and most studies were carried out in an emerging country, albeit in specialized centers. Such fact reinforces that in appropriate places, telesurgery is feasible and may be stimulated. With the aim of providing better practical guidance, the best experts and representatives in the field came together at the First Telesurgery Consensus Conference in the United States in 2024. During the meeting a joint effort to expand the legal, ethical and financial challenges was evident and other platforms showed their interest and advances in telesurgery, such as Hinotori, Edge, Kangduo and Microport Medbot (21).

Although there are restrictions to the use of robotic platforms such as cost, specialized training, learning curve, available technological resource, appropriate material, several trials started demonstrating the possibilities of performing remote surgeries. The first urological telesurgical procedure was carried out approximately 26 years ago using a PAKY model to perform a percutaneous anal access with success (22, 23). Searching for positive results in order to prove the safety of this modality and enable it to evolve, pre-clinical trials were carried out to test several procedures (24, 25). Zheng et al. performed 4 long distances laparoscopic surgeries in pigs. Although the sample accounted for only 50% of urinary tract surgeries (one nephrectomy and one cystectomy), Microhand platform and 5G technology were used and there was a mean network delay of 264 milliseconds during procedures with no complications (26). In 2023, Chu et al. used flexible ureteroscopy to fragment kidney stones with FURS robotic system remotely and transatlantic, more than 2,300 kilometers away from the operating room (27). Nguan et al. presented 18 robotic pyeloplasties, remotely with the Zeus platform, using IP-VPNe and via satellite, both successfully (28). Fan et al. implanted a Double J catheter, using wireless network and 5G technology with mean latency time of 272ms, with no complications (25).

There is evidence that the post-operative period can be maintained remotely enabling greater patient acceptance of the procedure. A randomized trial followed 270 patients who required hospital stay up to 72 hours comparing groups that received traditional face to face visits and groups with remote visits. The identified outcomes were similar as to morbidity rates, hospitalization, sick patient satisfaction and complications which demonstrated that virtual visits were not worse when compared to traditional face-to face model (29).

The network mode of operation utilized plays an important role in providing secure data transmission and 5G technology has its place in the spotlight although some configurations can achieve better results than others (30, 31). Aiming at only urological telesurgical procedures in humans, these studies were not included in our sample, even so, the results found in this research were compatible with the literature showing the 5G connection technology as the most widely used today (98.14%) in urological telesurgeries.

Improvement programs have been developed demonstrating that it is possible to enhance the urologist's performance in robotic surgeries (32). Remote diagnosis, tele -mentoring, live surgeries transmission, tele-training and tele-assistance, have gained room to quality education and have been used by many professionals, enabling the surgeon to become familiar with this technology (33–36). A randomized trial compared a percutaneous renal puncture performed by on-site urologists to another group carrying out the procedure controlled by a transatlantic remote robot, showing that, although the robotic puncture was slower, it was more precise and needed a smaller number of primary punctures in order for the procedure to be successful (37). The learning curve in telesurgery is continuous with the help of "medical surgical proctories", aiming at improving the quality of life of the professionals involved, optimizing costs and eliminating long periods of transportation, being able to allocate their time to other activities such as remaining closer to their families, being able to study and have leisure time, among others. Another interesting vision is the possibility of non-remote robotic surgeries being "converted" into remote ones in significantly intraoperative complications, or whenever it demands help from a more experienced professional.

There are reports of meticulous surgeries being carried out such as nephroureterectomy with as efficient results as traditional platforms (38). In this context, robotic platforms are constantly evolving to offer the surgeon the best tools, as it has been mentioned about the tactile sensitivity (24). A recent update of "Da Vinci" from Intuitive Surgical ^®^ has made this resource available. Despite that, other companies are searching for their market share such as KangDuo Surgical Robotic^®^, Edge Medical Robotic^®^, Versius^®^ from Cambridge Medical Robotics, and Hugotm System, RAS from Medtronic^®^, demonstrating their qualities, their benefits and soon they will be available at more affordable prices. Moschovas et al. performed a telesurgery robotic-assisted radical prostatectomy using Edge Medical Robotic^®^ in a 71-year-old patient in only 60 minutes without complications and excellent patient evolution, walking in just 4 hours after the procedure (39). Because the date when this case report was published exceeded our research period, it was not included in our sample.

In a secondary analysis we identified that MicroHand S was the most used platform, accounting for 83% of the procedures, corroborating with the arrival of new robotic systems. Due to the great potential of telesurgery, demonstrating such data may alert other companies about the concentration of this modality in only one platform, fomenting new research and new projects.

More complex procedures such as partial nephrectomy and radical cystectomy, were also described as with no complications confirming how safe this modality can be. Wang et al. performed a right partial nephrectomy in approximately 48 minutes, with estimated bleeding of 10 mL, with no complication and hospital stay of 4 days 4 (18). Yang et al. performed a remote radical cystectomy with left urethrectomy in 5 hours and cutaneous exteriorization of a ureteral stoma in a 71-year-old patient, with intraoperative bleeding of 200 mL, with a mean network delay of 254ms, without intraoperative complications (19). Analyzing the subgroups, we found that nephrectomy was the most remotely performed procedure, although it is not a complex procedure, relevant complications were not identified in these patients. Also, surgical treatment of varicocele was performed by telesurgery through a 5G network with a mean delay of 130ms, with minimal bleeding and finally a renal cyst excision, both uneventful with excellent results (15, 20).

Total latency time may be obtained through network latency with enough time for the robot to process the sign and perform movements, however, although they are similar to the values described, they can be discordant. Acceptable values to use 5G technology to carry out procedures with favorable surgical performance vary between 300 and 330 milliseconds, and the ideal ones are below 200 or 300 milliseconds (40, 41). Xu et al. proposed a network latency grading and showed that the impact is considered mild when latency values are ≤ 200 milliseconds, big when they are between 300 and 700 milliseconds and very big between 800 and 1000 milliseconds (41). As demonstrated in Table-1, total latency time in this review with 54 telesurgeries was 176 (IQR) 10.9 milliseconds. Although it is limited data, it strengthens the hypothesis that telesurgery is a viable mode. Despite the slight divergency, it is known that the greater the latency, the greater the likelihood of compromising the quality of the surgeon's movements.

Another important report is that there may exist a significant increase in the number of satellites orbiting the earth, which may reach up to a million of them (42). The combination of all these factors is a proof that within the telecommunication market there will be a relevant competition among companies, in such a way that technological improvement and lower costs will be inevitable, making access more affordable and driving the evolution of telesurgery.

There is evidence showing that not only remote surgeries can take place, but also tests can be carried out remotely. Despite its limitations, tele-cystoscopy was performed demonstrating that it can be done remotely (33). Tele-ultrasonography was not different. It was evaluated and found to be of diagnostic value in an intensive care unit, confirming that it is possible to identify kidney pathologies remotely (35). Therefore, tests carried out remotely may benefit many people who wait in endless queues to be attended in less favored areas.

The evolution of telehealth services has shown significant growth, exponentially, and can reach an increase of 235% per year (43). Turning our attention to the modality of telesurgery, despite being underutilized, the interest in monitoring its development has been gaining ground among urologists, since, despite the cost of the transmission equipment may reach US$70.000, such technology can be sustainable and generate huge return for the global health system (44). Although the cost of a robotic system is high and can reach 1.7 million, it is an abundant market with a potential financial turnover of US$ 5 billion (45). Since its implementation may cause a significant socioeconomical impact and influence policies for the distribution of health capital, a consideration that may seem like an insult, although very relevant, must be discussed, because this money can also be directed to other modalities that could benefit patient care (44).

Each patient must have their autonomy preserved, maintaining their right to decide based on their reasons and motives. Ethical aspects are seriously involved, such as, the risk of dehumanization, objectification of the patient, restriction of emotional connection and empathy, physician-patient relationship, medical assistance, as well as feeling satisfied with their expectations of care. That is the reason why, international guidelines and protocols need to be better established. Another aspect is that, in case of an eventual conversion to an open procedure, less and less likely these days, the local assistance team may not have the surgical expertise compared to the remote surgeon. In order to meet the patient's expectations, an informed surgical consent with precise guidance about the whole process must be discussed (46–48). In Brazil, telesurgery has been regulated by the Federal Council of Medicine (CRM) by means of resolution n° 2.311/2022, published on March 22, 2022 (48).

Although the initial investment is considerable, although sustainable, in the long term telesurgery can reduce healthcare costs in lower income countries reducing length of hospital stay, blood transfusions, surgical and nosocomial inflections and consequently, less use of antibiotics and fewer resistant strains, higher bed turnover and early return to the patient's productive working life (49). Other benefits would be to improve the training of examinations and procedures, reduce waiting lines, optimize the time of surgical instructors, support surgeons in more complex surgeries and to avoid intraoperative complications.

Randomized trials with robust samples were not found, nor trials comparing urological telesurgeries in humans. It is known that reports of a series of cases are limited, as well as the utilization of "salami slicing", where data of a fully complete sample are not identified, there is a risk of several types of biases and may represent outcomes that are not consistent with the population to be studied. Our critical assessment indicated that the majority of the trials are limited, however, the topic in question is promising and of high scientific relevance. Another important aspect worth emphasizing is that estimates demonstrate an inefficient number of surgeons in relation to the demand for the next 10 years, since low-income countries correspond to a volume which is approximately 50% of the world's population and approximately only 20% of all available surgeons (35, 50). Therefore, it is justified to draw the attention of these professionals and organizations involved in this innovative technology and to stimulate the production of new trials and discussions on the subject since its inclusion in the surgical routine will be inevitable in a very near future.

## CONCLUSION

Despite its limitations, we found evidence that performing robotic surgeries in the genitourinary system is feasible and safe, however further studies should be carried out. Telesurgery is presented as an innovative, promising modality and that is why, in a near future it may become a reality in many surgeon's practices.
